# Think You Can Shrink? A Proof-of-Concept Study for Men’s Health Education Through Edutainment

**DOI:** 10.1007/s41347-016-0009-8

**Published:** 2017-01-11

**Authors:** Thomas Ungar, Cameron D. Norman, Stephanie Knaak

**Affiliations:** 1 Department of Psychiatry, North York General Hospital, Toronto, ON Canada; 2 Faculty of Medicine, University of Toronto, Toronto, ON Canada; 3 Cense Research + Design, Toronto, ON Canada; 4 Dalla Lana School of Public Health, University of Toronto, Toronto, ON Canada; 5 Mental Health Commission of Canada, Ottawa, ON Canada

**Keywords:** Men’s health, Health education, YouTube, Internet, Webcasts, Mental health, Proof of concept study

## Abstract

Connecting people to useful, actionable health resources is a substantive challenge that sits at the heart of health communication. Digital media provides means of producing, distributing and revising content and creates possibilities for new and multiple channels for reaching and engaging audiences, particularly when combined with social media. While there is much promise of digital media forms to deliver audiences and promote engagement, the health communication landscape is still largely hit-and-miss with few ‘best practice’ examples to follow. Proof-of-concept studies allow for a structured, focused exploration of ways to leverage the potential of digital media and learn what approaches have the promise to invest resources in amid a sea of possible options. Think You Can Shrink? (TYCS) is a multi-episode web series modelled on a reality TV show format. The show’s key objective is to educate men and demonstrate, through modelling, ways men can support other men to encourage help-seeking behaviours and greater health communication, which in turn, may also lead to better health outcomes. Given the newness of the approach, the project was launched as a proof-of-concept study to explore: (a) whether this approach could engage the interest of men, (b) what initial impact this approach might induce and (c) the kind of audiences this approach might most appeal to.

## Introduction

Connecting people to useful, actionable health resources is a substantive challenge that sits at the heart of health communication. Digital media provides means of producing, distributing and revising content and creates possibilities for new and multiple channels for reaching and engaging audiences, particularly when combined with social media. While there is much promise of digital media forms to deliver audiences and promote engagement, the health communication landscape is still largely hit-and-miss with few ‘best practice’ examples to follow (Hayes et al., 2016) except for the calls to tailor content to audiences rather than aiming for mass appeal (Hayes et al., 2016).

Digital media enables health promotion to discover new audiences and engage hard to reach groups through a variety of means and mobile considerations (Bennett & Glasgow, 2009; Norman, 2012; Norman et al., 2016) with each having its own distinct design considerations. As these technologies and the algorithms supporting them co-evolve, understanding what tools hold potential to support health promotion and decision-making through proof-of-concept studies to full implementation trials, especially with groups who are often difficult to engage, becomes critical to ensuring resources are used wisely.

Think You Can Shrink? (TYCS) is a multi-episode web series modelled on a reality TV show format. The premise of the series is that men with no professional counselling experience play the role of contestants who seek to help another man (played by a standardized patient) with a specific condition or problem. Although the reality TV show format is not new in and of itself, the combination of web-based, long-form media with high production value with a reality TV format to focus on male health issues is entirely new. It was not clear whether this approach could even work, so a proof-of-concept study (Rabinowitz et al., 2013) was designed to explore whether this approach engage the interest of men, what initial impact this approach might induce and the kind of audiences this approach might most appeal to. Results can be used to provide guidance for further development of the intervention and future studies.

Given the rapid evolution of information technology, relying on full-scale evaluations to inform action is often impractical given the lag time between implementation and evaluation and the large number of techno-social variables that combine together in any particular intervention. Proof-of-concept studies allow the ability to generate evidence that a particular treatment, tool, or approach is feasible, effective and provides added value over existing approaches (Rabinowitz et al., 2013). Often, proof-of-concept studies are focused pilot trials that help determine whether larger, more definitive studies are indicated and guide intervention evolution appropriately (Rabinowitz et al., 2013). They are critical for testing out new ways to address complex problems where evidence is not available due to the novelty of combinations, but where need and potential reach or impact may be high. Thus, by taking a proof-of-concept approach, the TYCS project was implemented and tested as a prototype to explore the kinds of outcomes that may be generated and could be assessed in a full evaluation of the intervention in naturalistic conditions (e.g., see (Norman et al., 2016)).

Men, particularly young men, are much less likely than women to seek help for a range of health and mental health issues (Emslie et al., 2007; Fish et al., 2015; Haddad, 2013; Oliver et al., 2005). Men are also particularly vulnerable to depression and less likely to seek out support when confronted with mental health challenges (Addis, 2008). Suicide rates are four times higher for men than for women, and suicide remains one of the top three causes of death for men between the ages of 18 and 44 (Canadian Institute for Health Information, 2012; Statistics Canada, 2011). While a 2005 review by Galdas et al. (2005) of men’s help-seeking behaviours found substantial gaps in our understanding of the various factors that facilitate and hinder men’s propensity to give or receive help for a health problem, there are some important factors we do know about. For example, embarrassment and stigma (Cramer et al., 2014), perceived personal weakness (non-masculine behaviour) (Addis & Mahalik, 2003), as well as a lack of knowledge about health risks (Galdas et al., 2005) have all been identified as barriers to help seeking. One area in particular that requires attention for mental health promotion is that of stigma, which has been recognized as a primary barrier to help seeking and openness (Corrigan et al., 2014; Thornicroft, 2008). This includes both the problem of self-stigma, as well as perceived stigma from others (Corrigan et al., 2014; Thornicroft, 2008).

One of the most promising strategies for reducing the stigma of mental illness is that of contact-based education—meeting a person with a mental illness who is in recovery, either in-person or virtually—to learn about their experiences and to allow for a personal connection to be made (Corrigan et al., 2012; Tropp, 2009). Also, a recent paper recommends the use of human-centred design approaches, which take into account for important factors such as cultural context and unconscious biases in intervention design as a promising tool for effective anti-stigma interventions (Ungar et al., 2015). Hodgetts and Chamberlain (2002) found that television was an acceptable and unthreatening medium for men to explore sensitive health issues, when given the opportunity to witness modelling and positive examples of men seeking and receiving help. And Demyan & Anderson (2012) found that brief media interventions can have positive impact on mental health help seeking.

## Methods

### Project Implementation

The TYCS project revolves around a reality TV show format for health promotion and education using an ‘edutainment’ approach. Contestants, everyday men without counselling training, seek to help a ‘patient’ played by a standardized patient. A judge’s panel consisting of a psychiatrist, a family doctor/emergency room physician and a professional/celebrity interviewer critique a contestant’s performance after the fact. Episodes were created for a range of physical (testicular lump and prostate problem cancer fears) and mental (manic behaviour, OCD, depression and anger management) health issues. The show’s key objective is to educate men and demonstrate, through modelling, ways men can support other men to encourage help-seeking behaviours and greater health communication, which in turn, may also lead to better health outcomes. The TYCS series included six episodes and an original theme song hosted on YouTube as part of a channel (https://www.youtube.com/user/KathyMorePls) and embedded within a project website (http://www.thinkyoucanshrink.com/). Eight (Haddad, 2013) short preview (teaser trailer) videos were also made available via the YouTube channel.

Men over the age of 18 who had not had any counselling training were recruited to participate in the project by responding to a reality TV-style casting call by a professional casting agent. Participants had to consent to being part of the show knowing that it would be distributed via YouTube and possibly other means as well (e.g., broadcast television, on-demand services). Participants were informed about their role (i.e., providing supporting to another man experiencing a health concern) but were not told in advance the specific topic they would be discussing nor were they provided any advice on how to approach the situation. Episodes were staged and filmed in a hotel room with a film crew as well as the principal investigator/innovator (a registered psychiatrist). All participants were debriefed following their participation.

Videos were edited for length and included an expert commentary, which was filmed separately. Videos were uploaded to a TYCS YouTube channel and embedded on the TYCS project website. The videos were uploaded in September 2015. Resources for promotion were limited. As such, the TYCS series relied heavily on word of mouth and users discovering the videos through different means. Additional campaign promotion activities included social media, presentations at professional and public education events, as well as the PI’s own professional mental health networks. The promotional emphasis was on encouraging viewers to share and distribute links to the videos. Promotional work was done largely in Fall and Winter 2015/2016.

### Data Collection

For the purposes of this study, the cycle of data collection was 1 year to enable a detailed look at the longitudinal patterns of use and interest. The intent was to explore general perceptions of viewers and capture response behaviour related to an embedded survey to consider whether the method was viable. Data were collected through two main means. (1) Youtube and website use statistics: YouTube features built-in tools to track visits to a video, viewing patterns, visitor demographics and the playback—devices used. Standard web-use statistics such as viewing behaviour (e.g., amount of views and length of view), hardware and software used, visitor demographics (when available) including country of origin and referral sources were captured. (2) A structured, web-based evaluation survey: A 12-item survey delivered via an embedded web link on the TYCS website and YouTube. The survey included demographic questions and exploratory questions on learning outcomes, behavioural intentions (e.g., likelihood of employing listening techniques, opinion on format, message perception, current use of social media and Net Promoter Score). The Net Promoter Score asks how likely a person is to recommend the product to another and is considered to be a key measure of consumer loyalty and satisfaction (Reicheld, 2003). The survey was secure and anonymous and was entirely voluntary.

### Findings

#### User Statistics

The length of the videos ranged from 18:40 to 34:48 min (mean = 26.67 min). The average (mean) number of views per video was 340 (range = 36–700), and the percentage of videos viewed ranged from 19.6% to 34%. Fifty-three per cent of the views were on desktop computers (average viewing time 5.7 min), 36% were on mobile devices (average viewing time 5.3 min) and 9% were on tablets (average viewing time 6.5 min). Most of the audience came from Canada (2286 views), followed by the USA (249), UK (65), Australia (57) and Bulgaria (25). When audience location was examined by average duration per view and percentage of videos watched, Ukraine (10.3 min/44%) and Australia (8.1 min/25%) had the longest viewing averages and percentages of videos watched.

Audience traffic came mostly from within the YouTube ecosystem (55% of all views) with the rest coming from external sources such as the project website or social media. Among those accounts that had verified ages associated with them, the audience age distribution was as follows: 18–24, 11% of views; 25–34, 48% of views; 34–44, 22% of views; and 45+, 19% of views. Of those accounts that had a verifiable gender associated with them, 67.5% were male accounting for 60% of the views and 32.5% identified as female accounting for 40% of the views.

The TYCS project website itself had 1025 visits with an average of 2.54 page views per visitor. The majority of the non-search-related referrals to the TYCS website came from Facebook (*N* = 86, 58% of total), both mobile (*N* = 48, 33% of total) and the regular site (*N* = 38, 26% of total). Of the page views, 115 (5% of total) included visits to additional resources for men’s health.

#### Evaluation Survey

Forty-four individuals completed the survey in its entirety with no partial completions among the sample. Fifty-five per cent of the respondents were male (*N* = 24), 37% were female (*N* = 17) and three selected the option to not answer. Monthly social media use was distributed across the main platforms, with YouTube (*N* = 32, 73% of respondents), Facebook (*N* = 31, 71%), LinkedIn (*N* = 24, 55%) and Instagram (*N* = 21, 48%) serving as the most used social media tools among the options. Only two respondents reported not engaging social media on a monthly basis.

The Net Promoter Score value was 23, which is slightly positive. This includes 7 detractors, 19 neutrals and 17 promoters among the responses. All but one participant reported finding Think You Can Shrink somewhat or very entertaining. Table 1 shows the results of the call-to-action questions, which asked participants to indicate whether they were more likely to engage in certain activities after having watched TYCS. No differences were detected by gender or age. Favourable responses to the two Likert scale questions regarding behavioural intentions and learning outcomes were as follows:

- After watching Think You Can Shrink, I feel more comfortable in supporting a friend or family member who had the same health issue as the client in this video (*N* = 38 agree or strongly agree, 86% of respondents)
- After watching Think You Can Shrink, I am more likely to seek help if I needed it (*N* = 33 agree or strongly agree, 75% of respondents)

**Table 1:** Call-to-action item responses

• After watching Think You Can Shrink I am more likely to	• Responding ‘Yes’ frequency (%)
• Seek professional help when I need it	• 14 (32)
• Talk to others when I feel down or angry	• 16 (36)
• Listen to others when they feel down or angry	• 21 (48)
• Recommend that people seek professional help when they need it	• 27 (61)
• Share the video with others I think could benefit from it	• 22 (50)
• Seek ways I can better support others with mental health professionals	• 10 (23)

Responses to the open-ended question posed at the end of the survey asking for additional comments and reflections are presented in Table 2. Thirteen participants (30% of respondents) volunteered comments.

**Table 2:** Viewer comments and reflections

• Positive comments	• Neutral/critical comments
• The judges were excellent—would be good to see them talk to the shrink wannabes directly—excellent host—impressive work for sure—good luck	• I am mortified at how easy it is to bungle a mental health situation. We need to lessen the stigma of mental health issues so that more people get the right help.
• Very entertaining and informative!	• Idea is good but not sure if its entertainment or educative.
• Great SP! Nice vehicle to make the point that ‘shrinking’ aka psychiatric work is not just being a good listener or a nice person. Excellent depictions of real-life situations. Really great for students. Would be great to have a geriatric mental health issue. Loved Anthony. Loved the concept and idea behind the show. Hope they continue to promote this to the youth and our society. It is very important information.	• As a person with lifelong depression, this was incredibly difficult to watch. I had to skip through much of the aggressive character’s section because his character was so arrogant. Having an entertainment news host as a judge took away from the message. Need to watch for body language.
• Excellent way to reach out to people in need by advocating for mental heath awareness.	•
• So original. Have not seen anything quite like it before. A new genre. Awesome!	•
• Great program	•
• Fabulous! What about some videos on … workplace stressors (adult bullying)	•
• I loved this Dr. Ungar. What a creative idea. And anything that would convince a man to go into therapy is a huge force for positive change in this (still) rather mysogynist culture. Bravo!	•
• Thank you for creating these videos. I have been ‘nagging’ my partner (42-year-old male) for months to go see a doctor about some persistent health issues. He did not know the last time he saw a GP, nor did he remember ever having a physical. I shared the video about the lump on the client’s genitals. His hesitation to seek help out of fear of the unknown really resonated with my partner, who booked a doctor appointment shortly thereafter. I think it helped my partner to know that this hesitation is perhaps typical of male health care avoidance and encouraged him to seek help. Thanks again. Very, very well done, informative and entertaining, which is a rare combination. I have already passed the link for the show on to a friend.	•

## Discussion

Think You Can Shrink introduced the idea of using a professional-quality, long-format, reality show format to reach men about matters of mental health. An explicit sense of what kind of impact it might have or what kind of audience it would attract could not be firmly established a priori, which is why a proof-of-concept approach was taken for this project. Used data indicate that many viewers found the series through YouTube, suggesting that it alone can be a vehicle to drive traffic, particularly if fully optimized as part of an intervention strategy. Data from the evaluation survey also provided useful feedback as it provided insight into elements that users found useful, memorable, interesting and worth further exploration in future developments although the results from the survey must be interpreted with caution given the low response rate. User statistics and survey feedback also suggest that viewers found the realty show format engaging, despite being much longer than what is recommended by marketers, which is typically 3 to 5 min. While short-format videos were initially considered, these shortened edits were ‘just not working’ from a creative standpoint and seemed to lessen the power of the narrative and the investment in the characters. Therefore, the PI and creative team decided to keep the episodes at their full length.

The choice to use YouTube as opposed to a private site was a practical one: it reflects real-world conditions given its popularity and the fact that over half of the views were generated from within the YouTube ecosystem through playlists, recommendations and other links justifies the decision. The disadvantage of YouTube is that it sacrifices the ability to monitor use traffic more precisely as the variety of analytical tools available and formats for collecting additional data are reduced. Some data are not consistently collected because many viewers to YouTube do not have accounts, leading to incomplete data on demographic variables such as age and gender. As well, any initiation of a video indicates a ‘view—aggregate statistics do not account for dropped connections, hardware or software failures or mistaken clicks or viewers re-watching episodes. Thus, the true denominator is difficult to assess. This also poses limits in extrapolating findings from the evaluation survey, which requires users to finish the video to engage with. Future studies may need to explore ways to get data that is deeper within population groups as well as broader in representation, overall.

The strategy behind TYCS was to leverage the joy of entertainment using reality TV as ‘the hook’ to firstly engage and entertain men and public audiences, and secondarily for it to provide a vehicle to educate and model to men ways to deal with mental health in hopes of reducing stigma and improving health communication and help seeking. By having standardized patient actors role-playing everyday conditions in a reality TV format supported with comments from the host and experts that the intervention could provide engaging, entertaining guidance on the ‘how tos’ of talking to and counselling a person to seek help for a specific health concern. The findings suggest there is an appetite for this kind of intervention and, among those who responded to the evaluation survey, there is an attraction and utility in using a creative, edutainment-style approach to engaging audiences.

Think You Can Shrink was an experimental vehicle to not only explore ways to engage audiences, particularly men, but also to explore who those audiences might be. Although perhaps the most obvious audience of interest is men in the public sphere, there has also been great interest among health professionals for the show and the TYCS model. The PI has been invited for numerous presentations to professional audiences to illustrate TYCS for potential use with clients/patients, as well as trainees and fellow colleagues. Even though the series was focused on men and men’s health, 40% of the gender-identified viewership were women. It could be that women are looking for ways to better support men or simply looking for ways to help others in general. It was also encouraging to note that the majority of views that could be age-identified were from the harder-to-reach groups of young and middle-aged men. Thus, the answer to the question of ‘who is TYCS for’ may simply be ‘many audiences’.

Additional feedback to the PI from several screenings resulted in another observation: many viewers wanted to know the singular purpose and intended audience for the show. Others commented that the ‘normalness’ or perceived accessibility of the psychiatrist on the judges panel made them feel more comfortable about seeking help. In other words, for some viewers, the reality show format had the advantage of disconfirming certain negative stereotypes or beliefs about mental health professionals—an unexpected stigma reduction-related consequence. These different kinds of viewer reactions open the consideration that perhaps the show has a multiplicity of effects in that it may represent different things to different people. In this way, TYCS may be somewhat of a ‘postmodern’ health promotion vehicle in that it fulfils multiple roles simultaneously and is not easily slotted in to a singular genre or category. The variety of scenarios combined with professional feedback in a compressed and engaging format offers something that is not available elsewhere. It provides a promising model of how experimental communication methods can be used as a means to attract multiple audiences simultaneously, which offers an opportunity to promote education and dialogue among patients and professionals simultaneously and is in keeping with the changing media landscape in spaces like Facebook and Twitter where messaging is available to everyone.
